# Risk Perception of COVID-19 as a Cause of Minority Ethnic Community Tourism Practitioners’ Willingness to Change Livelihood Strategies: A Case Study in Gansu Based on Cognitive-Experiential Self-Theory

**DOI:** 10.3390/ijerph20010292

**Published:** 2022-12-24

**Authors:** Wangbing Liang, Zhao Li, Yinggang Bao, Bing Xia

**Affiliations:** 1 Tourism College, Gansu Tourism Development Academy, Northwest Normal University, Lanzhou 730070, China; 2 Economics College, Lanzhou University, Lanzhou 730070, China; 3 Institute of Geographic Sciences and Natural Resources Research, Chinese Academy of Sciences, Beijing 100101, China

**Keywords:** tourism practitioner, risk perception of COVID-19, willingness to change livelihood strategies, risk attitude, livelihood capacity, cognitive-experiential self-theory

## Abstract

This study based on the cognitive-experiential self-theory, with risk attitude as the mediator and livelihood capacity as the moderator, explores the mechanism of the effect of risk perception of COVID-19 on minority ethnic community tourism practitioners’ willingness to change livelihood strategies. Taking 423 tourism practitioners from five minority ethnic tourism communities as the objects of investigation in Gansu Province, China. This paper empirically tests the theoretical model by using Amos and SPSS. The results indicated the following: Risk perception of COVID-19 has a significant positive impact on the willingness of minority ethnic community tourism practitioners to change their livelihood strategies. Risk attitude partially mediated the relationship between risk perception of COVID-19 and willingness to change livelihood strategies. Livelihood capacity negatively moderated the relationship between risk perception of COVID-19 and willingness of minority ethnic community tourism practitioners to change their livelihood strategies. Livelihood capacity also negatively moderated the mediation effect of the relationship between risk perception of COVID-19 and willingness to change livelihood strategies. Based on the research conclusions, it provides theoretical guidance and practical enlightenment for minority ethnic community tourism practitioners on how to improve the stability and sustainability of their livelihoods through the adjustment and transformation of livelihood strategies in the post-epidemic era.

## 1. Introduction

The COVID-19 pandemic has significantly impacted territories’ ability to receive tourists. This has led to changes in economic, cultural, social, administrative measures of administrative department and the development strategies of tourist destinations [1,2,3]. On the other hand, studies have shown that COVID-19 has triggered a wide range of travel fears, not only for tourists, but also for residents in tourist destinations [4,5]. People’s lifestyle has been forced to change, and research on the impact of the epidemic on the psychology and behavior of tourists and residents in tourist destinations has attracted more and more attention.

In recent years, with the development of tourism, the role of tourism in optimizing the livelihood structure and reducing the vulnerability of livelihoods has become more prominent, and tourism has become an important driving force for the transformation of the livelihood of tourism destinations [1]. The tourism industry has a low entry threshold and is sustainable from intergenerational transmission, which can improve residents’ economic income and related skills, provide more employment opportunities. Due to remote geographical location, low level of economic development, backward development concept and other reasons, the livelihood of the residents of the minority ethnic communities in Gansu has not been improved, with the deepening of tourism development, tourism is regarded as an important means to reverse the marginalization trend of the development of minority ethnic communities and plays a very positive role in improving the livelihood of minority ethnic minorities [6]. However, the outbreak of the COVID-19 at the end of 2019 has hit the tourism industry unprecedentedly. With the deepening of rural tourism development, ethnic community residents are increasingly dependent on tourism, in case of similar emergencies such as the COVID-19, which makes it difficult for the tourism industry to achieve sustainable development, the livelihood of tourism practitioners in ethnic communities will be greatly impacted, and the excessive dependence on tourism will increase the vulnerability of tourism practitioners’ livelihood [7]. In the past two years, with the arrival of the post epidemic era, although the epidemic has been basically controlled, it has not yet subsided. Small scale or even large-scale outbreaks may occur at any time, and the impact of the epidemic cannot be ignored [8]. Therefore, how to improve the stability and sustainability of the livelihood of ethnic community tourism practitioners through the adjustment and transformation of livelihood strategies under the impact of the epidemic has become a realistic problem faced by ethnic community tourism practitioners.

Livelihood strategy transformation is an important way to realize capital accumulation, improve livelihood level and enhance risk resistance [9]. Scholars have made many beneficial explorations on the influencing factors of livelihood strategy transformation, and most of the research on livelihood strategy transformation are based on the sustainable livelihood framework. Such as Shi Pengfei et al. (2021) studied the impact of livelihood capital and livelihood capacity on the choice of tourism livelihood strategies in ethnic regions based on the sustainable livelihood framework [10]; Liu et al. (2018) used a multinomial logit model to study the impact of farmers’ livelihood assets on their livelihood strategies in western mountain areas of China [11]; Yin Ke et al. (2020) studied the impact of differences in five types of livelihood capital of farmers in the Three Gorges Reservoir Area, namely physical, financial, social, human and natural on their livelihood strategies [12]. In addition to the objective influencing factors in the sustainable livelihood framework, some scholars began to try to study the impact of factors outside the sustainable livelihood framework on the transformation of livelihood strategies. For example, Sun Fengzhi et al. (2020) studied the impact of government trust on residents’ willingness to change their livelihood strategies [13]; Wang Junhan et al. (2020) studied the impact of relocation on the livelihood capital and livelihood strategies of poor households [14]; Su Fang et al. (2019) studied the impact of livelihood risk perception on livelihood strategies by taking the Shiyang River basin in China as an example [15].

The existing research has actively explored the influencing factors of the willingness to change livelihood strategies, but there are still some deficiencies. First of all, the existing research mainly focuses on the discussion of the influencing factors of the transformation of traditional livelihood strategies, and the research fails to effectively focus on the influencing factors of tourism-based livelihood strategies. Secondly, most of the existing studies on the transformation of livelihood strategies are based on the sustainable livelihood framework, while the dynamic and quantitative consideration of the impact of other subjective and objective factors outside the framework on the transformation of livelihood strategies is relatively lacking. Finally, most of the existing studies generally regard the community residents as a whole, and the epidemic has caused a huge impact on the livelihood of tourism practitioners. However, the existing studies rarely discuss the impact mechanism of the transformation of the livelihood strategies of tourism practitioners in ethnic communities based on the epidemic background. Whether the risk perception of COVID-19 of ethnic community tourism practitioners on the epidemic will affect their willingness to change their livelihood strategies. Specifically, for tourism practitioners in ethnic communities, whether risk perception of COVID-19 is a factor affecting their willingness to change their livelihood strategies, what are the mechanisms and boundary conditions that risk perception of COVID-19 affects the willingness to change livelihood strategies. These are topics worthy of further discussion in theory and practice.

The academic research on the intermediary mechanism of the willingness to change the livelihood strategy is mainly based on the sustainable livelihood framework and some other subjective and objective factors. The variables involved mainly include livelihood capital [10,11], residents’ participation [13] and livelihood capacity [9]. However, there is no research on the impact of risk perception of COVID-19 on the willingness of ethnic community tourism practitioners to change their livelihood strategies from the perspective of risk attitude. Risk perception of COVID-19 is the subjective feeling and understanding of residents in ethnic communities on the external objective risk of COVID-19 epidemic [16], it is also an important factor affecting the psychological state and beliefs of tourism practitioners in ethnic communities. Changes in the psychological state and attitude of tourism practitioners when they perceive the risk of epidemic disease will have an impact on their will and behavior [17,18]. The risk attitude expresses the degree of aversion or preference of tourism practitioners in ethnic communities to the uncertain results of their own behaviors in the face of the unstable factor of epidemic [19]. Therefore, this paper believes that the risk attitude of tourism practitioners in ethnic communities may play a mediation role between the risk perception of COVID-19 and the willingness of tourism practitioners to change their livelihood strategies. In addition, although the risk perception of COVID-19 and its impact on the risk attitude of tourism practitioners in ethnic communities can easily alter their willingness to change livelihood strategies, their impact on the results is often affected by individual characteristics and their own specific circumstances. Livelihood capacity refers to the ability of individuals to adjust passively to the fragile environment and actively deal with, utilize and create opportunities [20]. It is a relatively stable objective factor and reflects the individual characteristics and specific conditions of tourism practitioners in ethnic communities. Individuals with weak livelihood capacity are more likely to magnify the perceived risks in the living and working environment, and more likely to affect their risk attitude, thus affecting their behavior and decision-making. At present, there is still a blank on the effect of the livelihood capacity of tourism practitioners in ethnic communities on the risk perception of COVID-19 and the results. To make up for this deficiency, this study will discuss the moderation role of the livelihood capacity of tourism practitioners in ethnic communities in the perception of epidemic risk and risk attitude.

Based on the cognitive-experiential self-theory, this study attempts to explore the impact of risk perception of COVID-19 on the willingness to change livelihood strategies from the perspective of ethnic community tourism practitioners. By focusing on the subjective psychological perception of the threat of the epidemic situation and the objective livelihood capacity of tourism practitioners in ethnic communities, explore the internal mechanism of this impact process (the mediated role of risk attitude) and whether there is a role boundary (the moderated role of livelihood ability), it is expected to theoretically make up for the lack of research on the willingness to change livelihood strategies from the perspective of ethnic community tourism practitioners, and provide theoretical guidance and practical inspiration for tourism practitioners on how to improve the stability and sustainability of their livelihoods through the adjustment and transformation of their livelihood strategies in the post epidemic era.

## 2. Literature Review and Hypothesis Development

### 2.1. Risk Perception of COVID-19 and Willingness to Change Livelihood Strategies

The livelihood strategy refers to that ethnic community residents can obtain positive livelihood output by combining and using their own livelihood assets to achieve their livelihood goals such as improving their living standards and obtaining more employment opportunities, including production activities, investment strategies [21]. Scones (1998) divided livelihood strategies into four categories: expansion, migration, intensification and diversification [22]. According to the main income sources, labor input direction and livelihood methods of farmers, farmers’ livelihoods can be divided into six types: migrant oriented, semi-industrial and semi agricultural, pure farmers, tourism franchise, balanced part-time and tourism oriented [23]. The livelihood strategy in this paper refers to the participation of ethnic community residents in tourism as a major way of livelihood according to their own wishes and capital under the background of tourism development [24]. The corresponding willingness to change the livelihood strategy means that in the context of the epidemic situation, the residents of ethnic tourism communities adjust their previous strategy of participating in tourism as a major way of livelihood according to their own wishes and actual conditions, so as to achieve the optimization of the way of livelihood.

The COVID-19 has caused a major blow to the tourism industry, and the livelihood problems of tourism practitioners in ethnic communities who rely on the tourism industry have followed, in the face of the threat of epidemic, the risk perception of COVID-19 of tourism practitioners in ethnic communities is their most direct subjective perception and understanding of the epidemic [25], and changes in the risk perception of COVID-19 may then have an impact on their will and behavior. Zhao et al. (2022) found that risk perception of COVID-19 will have an impact on individual hoarding behavior [17]; Dang’s (2022) research based on the expanded planned behavior theory model found that risk perception of COVID-19 will have an impact on residents’ sustainable travel willingness [18]; Gainza Perez Ma et al. (2022) found that risk perception of COVID-19 will have an impact on individual suicide behavior [26].

The basic assumption of the cognitive-experiential self-theory is that individuals respond to external information through two systems: empirical system and rational system [27]. In the context of the COVID-19, individuals’ risk perception of COVID-19 is based on intuitive feelings and judgments about their vulnerability to infection, which, respectively, reflect a dual process theoretical model that takes into account emotional feelings (experience) and cognitive judgments (rationality), that is, when thinking about risk, individuals will simultaneously adopt a refined way based on rational analysis and a way based on subjective feelings, which is the core of the cognitive-experiential self-theory [28]. In the face of the threat of the epidemic, the tourism practitioners in ethnic communities’ risk perception of the epidemic are their most direct subjective feeling and understanding of the epidemic, that is, the response to the threat of the epidemic based on the experience system. A high degree of risk perception of COVID-19 will promote tourism practitioners to hold a relatively negative evaluation attitude towards their own situation, thus affecting their will and behavior [24,25,26]. The pressure and panic brought by the epidemic situation to tourism practitioners may cause them to reduce their contact with others because they are worried about infection, thus affecting their daily work and interpersonal relationships [29]. In the face of increasing regional restrictions, people may have a need to go out of their homes to contact nature, and a high degree of risk perception of COVID-19 may hinder the realization of this demand [30]. Additionally, if tourism practitioners believe that the epidemic cannot be controlled in the short term, they will take a more negative attitude towards their livelihood strategy of participating in tourism as a major way of livelihood, thus worrying about their future development [31]. Tourism practitioners judge the impact of the epidemic on themselves according to their perception of the external environment. They may adjust their current livelihood strategies to ensure their own safety and avoid risks out of fear of possible infection, fear of serious consequences caused by infection and negative attitude that the epidemic cannot be controlled in the short term. When tourism practitioners feel the threat from the epidemic, their willingness to change their livelihood strategies will encounter some uncertainty. In order to strengthen self-defense, tourism practitioners will deal with their own livelihood strategy issues in a more conservative way and try to avoid being at a disadvantage. Therefore, this paper proposes the following hypothesis:

**Hypothesis 1** **(H1).** *Risk perception of COVID-19 has a significant positive effect on minority ethnic community tourism practitioners’ willingness to change livelihood strategies.*

### 2.2. Mediation Effect of Risk Attitude

Risk refers to the uncertainty including loss and profit, and risk attitude refers to the degree of people’s aversion or preference to such uncertain results [19], that is, the attitude of tourism practitioners towards the uncertain result of whether they are willing to bear the risk brought by the epidemic. Existing research shows that individual differences, external environment and risk perception are the main influencing factors of risk attitude [32,33,34]. Under the same risk level, risk preference individuals and risk aversion individuals show different behaviors, that is, the level of risk attitude will have an impact on individual behavior and willingness [35], becoming an important antecedent variable to predict individual behavior and willingness.

This paper argues that risk attitude plays a mediation role between risk perception of COVID-19 and tourism practitioners’ willingness to change livelihood strategies. 

Firstly, risk perception of COVID-19 has a positive impact on risk attitude. In the actual judgment process, the risk attitude of individuals will change with different situations, and the occurrence of the epidemic has changed the environment of tourism practitioners in ethnic communities [36]. On the one hand, from the perspective of the internal environment, the outbreak of the epidemic may make tourism practitioners feel that their own and their family’s health is facing a greater threat, which may have a negative impact on their daily work and interpersonal relationships [37]. At the same time, the threat of the epidemic may aggravate the concerns of tourism practitioners about their own economic security, thus making them more sensitive [38]. These possible risks cause tourism practitioners to fear the epidemic and lead to the improvement of individual risk attitude. On the other hand, from the perspective of the external environment, the outbreak of the epidemic may make tourism practitioners feel that it is difficult to communicate with the outside world, obtain information and participate in public affairs, and it is more difficult to get help from relatives and friends when encountering difficulties, resulting in a sense of insecurity and tension, this psychological feeling may also be expanded subjectively by individuals, affecting their normal work and interpersonal relationships [39]. Based on this, according to the individual’s perception of their own environment formed by the perceived epidemic risk, tourism practitioners have a sense of insecurity and a sense of threat to the living and working environment, leading to the improvement of their risk attitude.

Secondly, risk perception of COVID-19 has improved the risk attitude of ethnic community tourism practitioners, thus further enhancing their willingness to change their livelihood strategies. The cognitive-experiential self-theory emphasizes that the experience system is one of two systems in which individuals respond to external information. The experience system is intentional and nonverbal. It is the result of automatic learning based on experience, and its operation is often outside of consciousness, based on the experience system, tourism practitioners will adopt an intuitive, emotion-based approach when thinking about epidemic risk [27]. On the one hand, when tourism practitioners perceive the threats and risks brought by the epidemic, they will have more concerns and fears. Considering the safety of themselves, their families, the loss and harm caused by the epidemic, tourism practitioners will face their own production and life with a higher degree of risk attitude. [40] On the other hand, the improvement of risk attitude makes tourism practitioners more cautious in their daily production and life, resulting in more social avoidance, and tourism cannot be carried out without extensive interpersonal activities [41]. Griffin et al. (2007) believed that when the environment became unsafe, individuals would reduce their efforts to make constructive changes to the status quo or current practices [42]. Due to the improvement of risk attitude, tourism practitioners are worried about the loss and harm caused by the epidemic to themselves and their families. Therefore, in order to reduce risks and challenges, tourism practitioners tend to adjust their livelihood strategies, leading to an increase in their willingness to change their livelihood strategies.

To sum up, this study believes that when tourism practitioners perceive the risks and threats brought by the epidemic, they will have more concerns and fears, leading to an improved risk attitude, which will make them more cautious in their daily production and life, and enhance their willingness to change their livelihood strategies. Therefore, this paper proposes the following hypothesis:

**Hypothesis 2** **(H2).** *Risk attitude plays a mediated role between risk perception of COVID-19 and willingness to change livelihood strategies.*

### 2.3. Moderation Effect of Livelihood Capacity

Although the risk perception of COVID-19 may lead to the improvement of the risk attitude of tourism practitioners in ethnic communities, due to the differences in the individual characteristics and specific conditions of tourism practitioners, different individuals have different perceptions and responses to the same adverse environment [43]. In order to further explore the boundary conditions that impact the risk perception of COVID-19 on the risk attitude of tourism practitioners, the livelihood capacity is introduced to explore its regulatory role on the above relationship. Livelihood capability refers to the ability of individuals to adjust passively to the fragile environment and actively deal with, utilize and create opportunities [20]. It may adjust the relationship between the epidemic risk perceived by tourism practitioners and the perceived results. Tourism practitioners can be classified according to their differences in livelihood capacity [44]: tourism practitioners with strong livelihood capacity have more stable livelihood strategies, stronger passive adjustment ability and active handling ability when facing risks and threats, and are insensitive to external risks and threats; tourism practitioners with weak livelihood capacity have weak ability to deal with risks and threats, and are more inclined to magnify the perceived risks in the living and working environment, thus tend to make a stronger response.

This study believes that livelihood capacity negatively moderated the relationship between risk perception of COVID-19 and risk attitude of tourism practitioners in ethnic communities. The risk brought by the epidemic is uncertain and difficult to control. Tourism practitioners’ risk perception of COVID-19 is highly subjective. Individuals with different livelihood capacity have different intensity of perception and emotional response to epidemic risk. Compared with tourism practitioners with strong livelihood capacity, tourism practitioners with weak livelihood capacity will become more sensitive when they perceive the risks brought by the epidemic, and they are more likely to perceive the potential threats to their own health, economy and life [45]. The basic assumption of the cognitive-experiential self-theory is that individuals respond to external information through two systems: empirical system and rational system [27], after perceiving the risks and threats brought by the epidemic through the experience system, that is, based on intuition and subjective feelings. Tourism practitioners will further analyze the actual threat of the epidemic to themselves through the rational system, that is, the analytical and refined way to assess their own livelihood capabilities. Although the characterization paths of these two systems are different, they both have adaptability and limitations. As long as one of the two systems fails, it will lead to individual adaptation problems [28]. Tourism practitioners with weak livelihood capacity are more inclined to make a strong response when they perceive the risks brought by the epidemic. Therefore, the negative disturbance brought by risk perception of COVID-19 is more obvious, and the risk attitude will be greatly affected. On the contrary, tourism practitioners with strong livelihood capacity are less likely to be aware of the potential threat to themselves and their families caused by the epidemic, thus reducing the risk of the epidemic and the impact of the threat on them. Their psychological security level is relatively weakly affected by the perception of epidemic risk. Therefore, this paper proposes the following hypothesis:

**Hypothesis 3** **(H3).** *Livelihood capacity negatively moderated the relationship between risk perception of COVID-19 and risk attitude of ethnic community tourism practitioners.*

### 2.4. Moderated Mediating Effect of Livelihood Capacity

**Figure 1:**
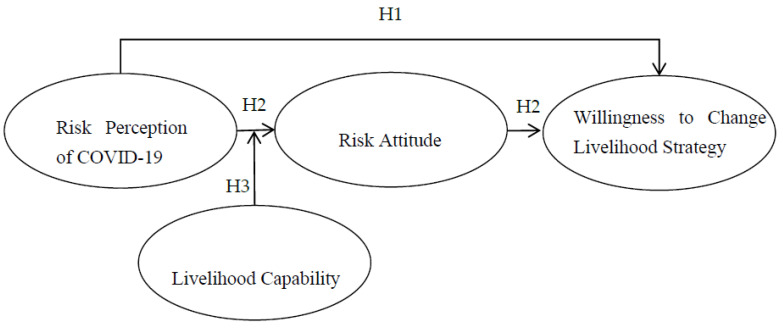
Conceptual model.

Combining H1, H2 and H3, this paper proposes a moderated mediating effect model (Figure 1). Research on risk attitude shows that individual differences, external environment and risk perception are the main influencing factors of risk attitude [32,33,34]. Livelihood capacity is one of the individual differences of tourism practitioners in ethnic communities. Tourism practitioners with weak livelihood capacity have obvious cognitive and emotional responses after perceiving the risks brought by the epidemic; therefore, their risk attitude is more vulnerable to the impact of risk perception of COVID-19, so the positive effect of risk attitude transmission of risk perception of COVID-19 on the willingness to change livelihood strategies may be enhanced. However, tourism practitioners with strong livelihood capacity have relatively weak cognitive and emotional responses when facing the threat of epidemic, and their risk attitudes are not easily affected by risk perception of COVID-19, thus weakening the transmission role in the positive relationship between risk perception of COVID-19 and willingness to change livelihood strategies. Therefore, this paper proposes the following hypothesis:

**Hypothesis 4** **(H4).** *Livelihood capacity negatively moderated the mediated role of risk attitude between risk perception of COVID-19 and willingness to change livelihood strategies.*

## 3. Methodology

### 3.1. Data Collection and Research Objects

This study collected data in the form of questionnaires. In order to ensure the reliability and validity of the questionnaire, the maturity scale in high-level journals was selected during the preparation of the questionnaire, and appropriate adjustments were made in combination with the specific research in this paper. The Likert Level 5 Scale was used for all items.

Specifically, for the measurement of risk perception of COVID-19, refer to the scale developed by Capone V et al. (2021), and measure from three aspects of severity, susceptibility and controllability [46], including six items: “COVID-19 infection will have a serious impact on health”; “There is a high possibility of death due to COVID-19 infection”; “I think the COVID-19 pandemic in my area is very serious and it is susceptible to infection”; “I feel that I will be more susceptible to infection COVID-19 than others”; “I think the spread of the COVID-19 is very difficult to control”; “I think COVID-19 is hard to cure”.

For the measurement of risk attitude, refer to the scale [19] prepared by EU Weber, which includes four items: “During the COVID-19 pandemic, I feel it is more difficult for me to get outside help”; “During the COVID-19 pandemic, I felt that my health and that of my family were facing greater threats”; “During the COVID-19 pandemic, I felt that I was facing greater economic risks”; “During the COVID-19 pandemic, I found it more difficult to obtain information and communicate with the outside world”.

The measurement of livelihood capacity is mainly based on the research of Burchardt [47] and Fukuda Parr [48]. It sets five questions from five aspects: health, education, access and use of services and information, political participation and social participation, including five questions: “The members of the family are all healthy”; “I have received skills training in tourism”; “My network communication and information acquisition are convenient”; “I can give my opinion on the public affairs decisions in the community”; “My relatives and friends offer to help when I encounter problems”. 

The measurement of the willingness to change the livelihood strategy is mainly based on the analysis and research of Rongna et al. (2022) and other scholars on the change of livelihood strategy [49], including four items: “During the COVID-19 pandemic, in addition to operating tourism activities, I will actively consider other ways of livelihood”; “During the COVID-19 pandemic, I think the current environment is no longer suitable for tourism activities”; “During the COVID-19 pandemic, I could not obtain satisfactory economic benefits from tourism activities”; “During the COVID-19 pandemic, I am no longer willing to work hard for the tourism development of the community”.

The research objects are tourism practitioners from five ethnic tourism communities in Gansu Province, China, namely Labuling Town, Sangke Town, Langmusi Town, Gaxiu Village in Gannan Tibetan Autonomous Prefecture and Bafang 13 xiang in Linxia Hui Autonomous Prefecture, the five tourism communities involved in the survey continuously promote the development of rural tourism based on rich natural resources and ethnic cultural resources. At the same time, the development degree and development mode of these tourism communities are different. Therefore, the conclusions and suggestions deduced from the data analysis of the above case sites are to a certain extent universal. Before the formal survey, a small-scale pre survey was conducted, and the opinions and suggestions expressed on the survey items were adjusted according to the respondents’ opinions and suggestions, making the survey items easier to understand. The research team conducted a formal survey on five tourism communities in August 2022 by means of field research and questionnaire distribution. The investigation was conducted from August 15 to August 30, and the investigation team was composed of six members. A small-scale epidemic broke out in Gansu in July, providing a very good practice scenario for empirical testing of the theoretical model of this study. The respondents are mainly tourism practitioners from ethnic tourism communities. For those with low cultural level who cannot fill in the questionnaire, the research team helps the respondents to complete the questionnaire by explaining the content of the questionnaire and asking questions orally. At the same time, through interviews with local government personnel, village secretaries and other figures, we can learn about the local economic profile and the current situation of tourism development. A total of 463 valid questionnaires were collected. On this basis, the researcher further eliminated the questionnaires with obvious filling errors and serious data missing, and finally obtained 423 valid questionnaires, with an effective rate of 91.4%.

### 3.2. Basic Sample Description

The number of women in the valid questionnaire was slightly higher than that of men, with 46.3% male and 53.7% female. The overall age is on the high side, with nearly half of the people over 46 years old. The majority of ethnic groups are Tibetans. The overall educational background is low, most of them are junior high school students or below. Most of the monthly income is between 2000 and 8000. This sample is in line with the basic characteristics of tourism practitioners in ethnic communities and is representative. See Table 1 for details.

## 4. Analysis and Results

### 4.1. Reliability and Validity Test

First, SPSS was used to test the reliability. The results showed that the standardized load coefficients of risk perception of COVID-19, risk attitude, livelihood capacity and willingness to change livelihood strategies were bigger than 0.6, Cronbach’s α were 0.765, 0.895, 0.788 and 0.873, respectively, which indicates that the reliability of the research data is great. Secondly, the arithmetic square root of the extracted value of the average variance of each major variable is bigger than the correlation coefficient between this variable and other variables, indicating that the major variables in this study have good discriminant validity. In addition, the extracted value AVE of variance of each major variable was bigger than 0.5, and the combined reliability (CR) of each variable was bigger than 0.7, indicating that each major variable in this study had good aggregation validity. See Table 2 for details.

### 4.2. Reliability and Validity Test

It can be seen from the correlation analysis that the statistical relationship between the variables is strong, which provides preliminary evidence for subsequent research. There is a significant positive correlation between risk perception of COVID-19 and risk attitude, a significant positive correlation between risk attitude and willingness to change livelihood strategies, and a significant positive correlation between risk perception of COVID-19 and willingness to change livelihood strategies. See Table 3 for details.

### 4.3. Hypothesis Test

#### 4.3.1. Test of Main Effect

The path coefficient and fitting index of the model are shown in Table 4. When risk perception of COVID-19 affects willingness to change livelihood strategies, the value of standardized path coefficient is 0.241 > 0, and this path shows a significant level of 0.01 (z = 5.594, *p* = 0.000 < 0.01), which indicates that risk perception of COVID-19 will have a significant positive impact on willingness to change livelihood strategies, and the hypothesis H1 is supported.

#### 4.3.2. Mediation Effect Test

**Figure 2:**
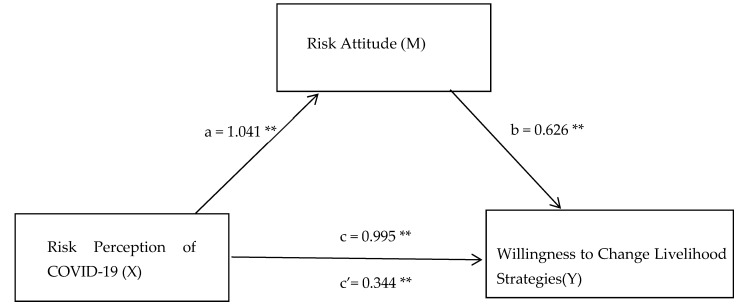
Mediation effects mode. Note: ** *p* < 0.01; a is effect of Risk perception of COVID-19 on Risk attitude; b is effect of Risk attitude on Willingness to change livelihood strategies; c’ is direct effect of Risk perception of COVID-19 on Willingness to change livelihood strategies; c is total effect of Risk perception of COVID-19 on Willingness to change livelihood strategies.

Firstly, when risk perception of COVID-19 affects risk attitude, the value of standardized path coefficient is 0.695 > 0, and this path shows a significant level of 0.01 (z = 17.856, *p* = 0.000 < 0.01), which indicates that risk perception of COVID-19 will have a significant positive impact on risk attitude. When risk attitude affects willingness to change livelihood strategies, the standardized path coefficient value is 0.657 > 0, and this path shows a significant level of 0.01 (z = 16.21, *p* = 0.000 < 0.01), which indicates that risk attitude will have a significant positive impact on willingness to change livelihood strategies, so the hypothesis H2 is supported. At the same time, Bootstrapping analysis technology is used to further verify the mediation effect of psychological security, as shown in Table 5. The mediation effect analysis involves three models, respectively: Willingness to change livelihood strategies = −0.232 + 0.995×risk perception of COVID-19; risk attitude = −0.412 + 1.041×risk perception of COVID-19; willingness to change livelihood strategies = 0.026 + 0.344×risk perception of COVID-19 +0.626×risk attitude. Table 6 and Figure 2 show that the direct effect of risk perception of COVID-19 of willingness to change livelihood strategies through risk attitude is 0.344 **, with a 95% confidence interval of [0.354, 0.569], excluding no zero. Therefore, H2 is assumed to be further supported. In addition, because the intermediary effect value is the same as the direct effect value, the sense of risk attitude plays a part of intermediary role between the risk perception of COVID-19 and the willingness to change livelihood strategies.

#### 4.3.3. Moderation Effect Test

**Figure 3:**
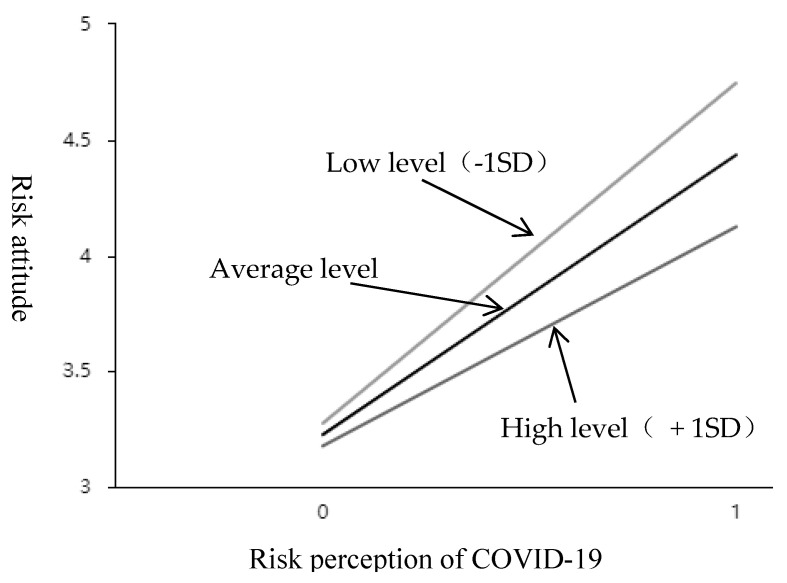
The effect of interaction between RP and LA on RA.

As shown in Table 7, the interaction item between risk perception of COVID-19 and livelihood capacity style presents a significant (t = −5.528, *p* = 0.000 < 0.05), and has a negative impact (β = −0.257, *p* < 0.05). It means that when the risk perception of COVID-19 affects the risk attitude, the moderation variable (livelihood capacity) has a significant difference in the extent of influence at different levels. See the simple slope diagram in Figure 3. Divide the scores of livelihood capacity style into two groups according to high (M + 1SD) and low (M-1SD). Perform a simple slope analysis. As shown in Figure 3, the top line represents the relationship between risk perception of COVID-19 and risk attitude under low livelihood capacity. The bottom line represents the relationship between risk perception of COVID-19 and risk attitude under high livelihood capacity. The middle line is the average level. It can be seen that the relationship curve between risk perception of COVID-19 and risk attitude of tourism practitioners is steeper and the slope is larger when the livelihood capacity of tourism practitioners is at a low level. When the livelihood capacity of tourism practitioners is at a high level, the relationship curve between risk perception of COVID-19 and risk attitude of tourism practitioners is smoother and the slope is smaller. The negative moderation effect of livelihood capacity was further tested, that is, H3 was further supported by empirical data.

#### 4.3.4. Moderated Mediation Effect Testing

As shown in Table 8, Bootstrapping method is used to analyze the intermediary effect of risk attitude between risk perception of COVID-19 and willingness to change livelihood strategies under different levels of livelihood capacity. The specific method is to add or subtract a standard deviation from the mean value of the livelihood capacity, divide the scores into two groups according to high (M + 1SD) and low (M − 1SD), get the values of the livelihood capacity at two different levels, and calculate the intermediary effect of risk attitude at these two different levels. For the intermediary variable of risk attitude, when the level is low, the boot 95% CI does not includes the number 0, which means that it has a mediation effect at this level, and the effect value is 0.920. At the average level, the boot 95% CI does not include the number 0, which means that it has a mediation effect at this level, and the effect value is 0.756. At a high level, the boot 95% CI does not include the number 0, which means it has an intermediary effect at this level, and the effect value is 0.593. To sum up, Boot LLCI and Boot ULCI do not include the number 0, and the change direction of the effect value is consistent with the change of the risk attitude level, indicating that they have a moderated mediating role. Therefore, H4 is verified.

## 5. Conclusions and Discussion

### 5.1. Conclusions

As an important livelihood strategy for ethnic tourism community residents, tourism is of great significance in promoting the restructuring, expansion and optimization of livelihood methods and improving the overall sustainability of livelihoods [50]. COVID-19 has posed a serious challenge to tourism practitioners in ethnic communities whose livelihood strategies are highly dependent on the tourism industry. How tourism practitioners improve the stability and sustainability of their livelihoods through the adjustment and transformation of their livelihood strategies in the post epidemic era has become a practical problem that needs to be solved urgently [51]. Therefore, from the perspective of tourism practitioners in ethnic communities, this study explores whether the willingness of tourism practitioners to change their livelihood strategies is affected by risk perception of COVID-19, and further identifies the role of psychological state (risk attitude) and personal characteristics (livelihood capacity). The findings are as follows: (1) Risk perception of COVID-19 has a significant positive predictive effect on ethnic community tourism practitioners’ willingness to change livelihood strategies. (2) Risk attitude plays a part of intermediary role between risk perception of COVID-19 and ethnic community tourism practitioners’ willingness to change livelihood strategies. (3) The livelihood capacity has a significant negative moderation effect on the relationship between risk perception of COVID-19 and tourism practitioners’ risk attitude. The stronger the livelihood capacity of tourism practitioners, the weaker the positive relationship between the risk perception of COVID-19 and tourism practitioners’ risk attitude. (4) Tourism practitioners’ livelihood capacity has a significant negative moderation effect on the indirect relationship between risk perception of COVID-19 and tourism practitioners’ willingness to change livelihood strategies through risk attitude. The stronger the livelihood capacity of tourism practitioners, the weaker the positive indirect relationship.

### 5.2. Discussion

#### 5.2.1. Theoretical Implications

Based on the background of the post epidemic era, this study confirmed the predictive effect of risk perception of COVID-19 on the willingness of ethnic community tourism practitioners to change their livelihood strategies. On the one hand, while focusing on the consequences of risk perception of COVID-19, existing scholars have ignored the impact of risk perception of COVID-19 on the willingness to change livelihood strategies from the perspective of tourism practitioners in ethnic communities. On the other hand, previous studies on the antecedents of the willingness to change livelihood strategies rarely involved the subjective factors of the objects represented by the community residents. Most of the studies were based on the sustainable livelihood framework, and few studies combined with the epidemic background to test the impact of the risk perception of COVID-19 of tourism community residents on their willingness to change livelihood strategies. In view of this, based on the questionnaire survey data, this study effectively expanded the research object to the tourism practitioners in ethnic communities with tourism as the main livelihood strategy, and further deepened the awareness of the relationship between the risk perception of COVID-19 and the tourism practitioners’ willingness to change livelihood strategies from the subjective factors affecting their willingness to change livelihood strategies. This study complements the research results between risk perception of COVID-19 and livelihood strategy change intention as one of the subjective factors of the object, and is also a response to the necessity of dynamically and quantitatively considering the impact of livelihood strategy change from other subjective factors outside the sustainable livelihood framework pointed out by Sun et al. (2020) [13]. This conclusion to some extent supports Li’s (2022) conclusion that risk perception of COVID-19 has a significant impact on consumers’ willingness to consume products from restaurants [52], and Zhao’s (2022) conclusion that risk perception of COVID-19 significantly and positively predicts individuals’ hoarding behavior [17]. It provides a theoretical explanation for the willingness of tourism practitioners in ethnic communities to change their livelihood strategies with tourism as the main livelihood strategy.

Second, the introduction of risk attitude as an intermediary has explored the mechanism between risk perception of COVID-19 and the willingness of tourism practitioners in ethnic communities to change their livelihood strategies, supplemented the content of the intermediary mechanism of livelihood strategy change through the path of perceiving individual internal motivation, which also responds to the proposal of X Lan et al. (2018) [53] that future research should explore residents’ adaptation to rural tourism as a new strategy. It is conducive to in-depth understanding of the impact of risk perception of COVID-19 on tourism practitioners. This research is analyzed from the perspective of cognitive-experiential self-theory, starting from experience system and rational system, it provides an effective analytical framework for exploring the mechanism of risk perception of COVID-19 on the willingness of tourism practitioners to change their livelihood strategies [27]. The risk perception of COVID-19 may cause tourism practitioners to have fears, cause stress and trouble on their spirit and daily work, and then improve their risk attitude, which will have a negative impact on their interpersonal relationships, and ultimately lead to the improvement of tourism practitioners’ willingness to change their strategies.

Finally, this study tested the moderation effect of livelihood capacity of ethnic community tourism practitioners. The level of livelihood capacity will affect tourism practitioners’ risk perception of COVID-19. Compared with individuals with strong livelihood capacity, individuals with weak livelihood capacity are more sensitive to the perception of epidemic risk, and will be more likely to perceive the potential threat of the epidemic to their own health, economy and life [54,55]; therefore, in the face of epidemic risk, the reaction is stronger, and it is easier to make negative feedback on the cognition of others and the external environment, these negative cognition and reactions will aggravate the positive impact of risk perception of COVID-19 on risk attitude. The risk attitude of tourism practitioners in their daily work environment will be improved more significantly, which will further promote the willingness to change their livelihood strategies. According to the cognitive-experiential self-theory, as a rational system for tourism practitioners to assess external risks and threats, livelihood capability will jointly influence their will and behavior with tourism practitioners’ risk perception of COVID-19 through the experience system [27,28]. On the one hand, this study provides empirical support for the interaction model of risk attitude of tourism practitioners. On the other hand, it also deeply explores the role boundary of livelihood capability in the willingness of tourism practitioners to change livelihood strategies, that is, the interaction between the livelihood capability, individual psychology and risk perception of COVID-19 in the willingness of tourism practitioners to change livelihood strategies. This research conclusion is an important supplement and extension to the research on the subjective factors of the object and the willingness to change the livelihood strategy.

#### 5.2.2. Management Implications

Empirical research shows that the willingness of tourism practitioners in ethnic communities to change livelihood strategies is affected by many factors. Therefore, to improve the stability and sustainability of the livelihood of tourism practitioners in ethnic communities through the adjustment and transformation of livelihood strategies in the post epidemic era, it is necessary to start from various aspects, enhance the adaptability of tourism practitioners, and reduce the risks and sensitivity of their livelihoods. This study puts forward the following suggestions based on the research conclusions and the actual situation of the case:

Firstly, from the perspective of the experience system of the cognitive-experiential self-theory, the risk perception of the epidemic situation by ethnic community tourism practitioners is the most direct subjective feeling and understanding of the epidemic situation, that is, the response to the threat of the epidemic situation based on the experience system. If there is no scientific and rational understanding of the epidemic, it may lead to excessive fear of the epidemic, which will lead to irrational behavior and response. Therefore, various measures should be taken to ensure that tourism practitioners have a correct understanding of epidemic risk, timely, comprehensive, objective and true report and notification of epidemic information, strengthen the popularization, publicity and education of epidemic knowledge, eliminate excessive worries and anxieties of tourism practitioners, so as to help tourism practitioners make rational decisions based on full and real information when facing the epidemic, scientifically and reasonably adjust their livelihood strategies to deal with the epidemic, and minimize the losses caused by the epidemic to tourism practitioners.

Second, from the perspective of the rational system of the cognitive-experiential self-theory, after perceiving the risks and threats brought about by the epidemic through the empirical system, that is, based on intuition and subjective feelings, tourism practitioners will further evaluate the actual threat of the epidemic to themselves through the rational system, that is, an analytical and refined way. The livelihood capacity of tourism practitioners is one of the important bases for their analysis through the rational system. Tourism practitioners with strong livelihood capacity have stronger passive adjustment ability and active handling ability when facing risks and threats, and are less sensitive to epidemic threats than tourism practitioners with weak livelihood capacity, which helps them respond to the epidemic with a more calm and rational attitude and behavior. Therefore, multiple measures should be taken to improve the livelihood of tourism practitioners. Introduce external excellent tourism operation training institutions to carry out tourism operation training. Perfect rural network communication and strengthen infrastructure construction. Improve the e-commerce skills of tourism practitioners, use new technologies and methods to promote the integration of online and offline businesses, and reduce the adverse effects of the epidemic to a certain extent. Establish a community tourism information sharing mechanism and create an information sharing platform. Guarantee work shall be well done to provide necessary support for tourism practitioners seriously affected by the epidemic, and improve the early warning mechanism for emergencies. Formulate and improve relevant policies to support the livelihood activities of tourism practitioners.

Thirdly, adopt appropriate tourism livelihood strategies that meet the market demand in the post epidemic era. In the face of increasing regional restrictions and the need to get out of the house and get in touch with nature, although COVID-19 pandemic has posed a serious threat to the livelihood of tourism practitioners in ethnic communities, market demand still exists [56,57]. Tourism practitioners should adopt appropriate livelihood strategies to stimulate tourists’ tourism desire as much as possible and improve the security of service process. On the one hand, the increase of regional restrictions makes local travel the first choice for tourists with a desire to travel [30]; therefore, tourism practitioners in ethnic communities should take the local and surrounding areas as the main target market, and strengthen the market publicity of the local and surrounding areas through community help and other ways. On the other hand, the COVID-19 pandemic makes tourists hope to reduce their contact with others as much as possible. Although this is unfavorable to traditional tourism and hotel industry, however, it makes minshuku, campground, caravan and other tourism methods popular [58]. Tourism practitioners can gradually adapt to the tourism market environment in the post epidemic era by adopting similar tourism livelihood strategies that minimize exposure, so as to improve the stability and sustainability of their livelihoods [59].

Finally, adopt diversified livelihood strategies to improve adaptability. The outbreak of the epidemic has once again proved that residents in tourism communities who rely too much on single life planning activities are highly vulnerable to livelihood, and are prone to unemployment and other problems when emergencies such as the epidemic occur. Therefore, tourism practitioners should adopt diversified livelihood strategies. Promote the diversification of income channels, and improve the ability to deal with various risks through various adjustment livelihood strategies such as savings, help from relatives and friends, loans from financial institutions, and migrant workers. Flexible choice of various livelihood strategies, such as tourism oriented, local labor and business, local diversified agriculture, dual employment, and out of town employment based on their own specific conditions, improve the sustainability and risk resistance of tourism practitioners’ livelihood, promote the restructuring, expansion and optimization of livelihood strategies, eliminate the adverse effects of the epidemic as much as possible, and promote the sustainable livelihood of tourism practitioners in ethnic communities to a higher level.

#### 5.2.3. Limitations and Suggestions for Future Research

First of all, this study takes the tourism practitioners of ethnic communities in Gansu as the research object, and the sample is concentrated in Gansu, which inevitably weakens the universality of the study. Future research can further expand the sample range to obtain more universal research results. Secondly, the impact of risk perception of COVID-19 on the willingness of tourism practitioners to change livelihood strategies is a complex process. This paper only considers the establishment of models from two aspects of risk attitude and livelihood capacity. Future research can introduce more variables to explore the impact mechanism of risk perception of COVID-19 on the willingness to change livelihood strategies from different theoretical perspectives. Finally, as a major public health emergency, the epidemic has a certain timeliness. Future research can compare the attitudes and behaviors of tourism practitioners before and after the epidemic, such as their willingness to change their livelihood strategies, to explore whether they will change over time.

## Figures and Tables

**Table 1:** Demographic characteristic.

Variables	Measurement Index	*n*	%
Gender	M	196	46.3
	F	227	53.7
Age	Under 18	5	1.2
	18–25	21	5
	26–35	76	18
	36–45	121	28.6
	46–55	126	29.8
	Over 56	74	17.5
Highest level of education	Junior high school and below	207	48.9
	Senior secondary/technical secondary and below	133	31.4
	Junior college	52	12.3
	College or above	31	7.3
Nation	Han nationality	96	22.7
	Tibetans	202	47.8
	Hui nationality	97	22.9
	Bai nationality	21	5
	Others	7	1.7
Monthly discretionary income	Under 2000 RMB	58	13.7
	2001–5000 RMB	131	31
	5001–8000 RMB	152	35.9
	Over 8000 RMB	82	19.4
Case	Labrang Town	101	23.9
	Sangke Town	64	15.1
	Langmusi Town	135	31.9
	Gaxiu Village	30	7.1
	Bafang 13 xiang	93	22

**Table 2:** Reliability and aggregation validity of the scale.

Dimension	Items	Std. Estimate	z (CR)	Cronbach’s α	AVE
Risk perception of COVID-19	Y1	0.688	0.71	0.765	0.523
	Y2	0.675			
	Y3	0.731			
	Y4	0.786			
	Y5	0.767			
	Y6	0.685			
Risk attitude	F1	0.895	0.878	0.895	0.706
	F2	0.882			
	F3	0.727			
	F4	0.845			
Livelihood capacity	S1	0.736	0.751	0.788	0.574
	S2	0.765			
	S3	0.749			
	S4	0.803			
	S5	0.733			
Willingness to change livelihood strategies	J1	0.705	0.869	0.873	0.626
	J2	0.771			
	J3	0.848			
	J4	0.832			

**Table 3:** Correlation coefficients and discriminant validity.

	Average Value	SD	Gender	Age	Education	Risk Perception of COVID-19	Risk Attitude	Livelihood Capacity	Willingness to Change Livelihood Strategies
Gender	1.535	0.499							
Age	2.561	1.11	0.033						
Education	2.915	0.881	−0.03	0.238 **					
Risk perception of COVID−19	3.616	0.729	0.009	−0.153 **	0.130 *	**0.723**			
Risk attitude	3.354	1.093	−0.02	−0.074	0.064	0.695 **	**0.84**		
Livelihood capacity	2.433	0.772	−0.008	0.185 **	−0.112 *	−0.673 **	−0.450 **	**0.758**	
Willingness to change livelihood strategies	3.367	1.041	−0.05	−0.065	0.134 *	0.697**	0.824 **	−0.387 **	**0.791**

Note: Diagonal bold values are square roots of AVE and values below the diagonal are Pearson correlation coefficients between dimensions; * *p* < 0.05; ** *p* < 0.01.

**Table 4:** Path coefficients.

X→Y	Non-Standardized Path	SE	z (CR)	*p*	Standardized Path
Risk perception of COVID-19→Risk attitude	1.041	0.058	17.856	0	0.695
Risk perception of COVID-19→Willingness to change livelihood strategies	0.344	0.058	5.942	0	0.241
Risk attitude→Willingness to change livelihood strategies	0.626	0.039	16.21	0	0.657

**Table 5:** Mediation effects model tests.

	Willingness to Change Livelihood Strategies	Risk Attitude	Willingness to Change Livelihood Strategies
Constant	−0.232 (−1.132)	−0.412 (−1.909)	0.026 (0.166)
risk perception of COVID-19	0.995 ** (17.927)	1.041 ** (17.806)	0.344 ** (5.914)
risk attitude			0.626 ** (16.134)
R 2	0.486	0.483	0.709
Adjust R 2	0.484	0.481	0.708
F	F (1340) = 321.390, *p* = 0.000	F (1340) = 317.044, *p* = 0.000	F (2339) = 413.406, *p* = 0.000

Note: T value in parentheses; ** *p* < 0.01.

**Table 6:** Mediation effects test results.

Path	c	a	b	a×b	a×b (Boot SE)	a×b (z)	a×b (*p*)	a×b (95% BootCI)	c’	Test Conclusion
risk perception of COVID-19→risk attitude→willingness to change livelihood strategies	0.995 **	1.041 **	0.626 **	0.651	0.055	11.772	0	0.354~0.569	0.344 **	Partial mediation

Note: ** *p* < 0.01.

**Table 7:** Moderation effect test results.

	Model 1					Model 2					Model 3				
	B	SE	t	*p*	β	B	SE	t	*p*	β	B	SE	t	*p*	β
Constant	3.354	0.043	78.786	0.000 **	-	30.354	0.043	78.716	0.000 **	-	3.226	0.047	68.685	0.000 **	-
Risk perception of COVID-19	1.041	0.058	17.806	0.000 **	0.695	1.075	0.079	13.575	0.000 **	0.717	1.209	0.08	15.165	0.000 **	0.806
Livelihood capacity						0.047	0.075	0.628	0.53	0.033	−0.063	0.074	−0.852	0.395	−0.045
Risk perception of COVID-19 * Livelihood capacity											−0.339	0.061	−5.528	0.000 **	−0.257
R 2	0.483					0.483					0.526				
Adjust R 2	0.481					0.48					0.522				
F	F (1340) = 317.044, *p* = 0.000					F (2339) = 158.437, *p* = 0.000					F (3338) = 125.024, *p* = 0.000				
△R 2	0.483					0.001					0.043				
△F	F (1340) = 317.044, *p* = 0.000					F (1339) = 0.395, *p* = 0.530					F (1338) = 30.564, *p* = 0.000				

Note: Dependent variable: risk attitude; * *p* < 0.05; ** *p* < 0.01.

**Table 8:** Conditional indirect effect test results.

Mediation Variable	Level	Level Value	Effect	BootSE	BootLLCI	BootULCI
Risk attitude	Low level (−1SD)	1.661	0.92	0.123	0.694	1.177
	Average level	2.433	0.756	0.105	0.569	0.976
	High level (1SD)	3.204	0.593	0.094	0.43	0.794

## Data Availability

The data analyzed in this paper are proprietary, and therefore cannot be posted online.
